# Relationship between the structure and composition of rumen microorganisms and the digestibility of neutral detergent fibre in goats

**DOI:** 10.5713/ajas.18.0043

**Published:** 2018-07-26

**Authors:** Kaizhen Liu, Lizhi Wang, Tianhai Yan, Zhisheng Wang, Bai Xue, Quanhui Peng

**Affiliations:** 1 Animal Nutrition Institute, Key Laboratory of Bovine Low-Carbon Farming and Safe Production, Sichuan Agricultural University, Chengdu, Sichuan 611130, China; 2 Agri - Food and Biosciences Institute, Hillsborough, Co. Down, BT26 6DR, UK

**Keywords:** Goats, Neutral Detergent Fibre Digestibility, High-throughput Sequencing, Rumen Microorganism

## Abstract

**Objective:**

This experiment was conducted to compare the structure and composition of ruminal microorganisms in goats with high and low neutral detergent fibre (NDF) digestibility.

**Methods:**

Nineteen crossbred goats were used as experimental animals and fed the same total mixed rations during the 30-day pre-treatment and 6-day digestion trialperiods. All faeces were collected during the digestion period for measuring the NDF digestibility. Then, high and the low NDF digestibility individuals were chosen for the high NDF digestibility group (HFD) and low NDF digestibility group (LFD), respectively. Rumen contents were collected for total microbial DNA extraction. The V4 region of the bacterial 16S rRNA gene was amplified using universal primers of bacteria and sequenced using high-throughput sequencer. The sequences were mainly analysed by QIIME 1.8.0.

**Results:**

A total of 18,694 operational taxonomic units were obtained, within 81.98% belonged to bacteria, 6.64% belonged to archaea and 11.38% was unassigned microorganisms. *Bacteroidetes*, *Firmicutes*, and *Proteobacteria* were the predominant microbial phyla in both groups. At the genus level, the relative abundance of fifteen microorganisms were significantly higher (p<0.05) and six microorganisms were extremely significantly higher (p<0.01) in LFD than HFD. Overall, 176 core shared genera were identified in the two groups. The relative abundance of 2 phyla, 5 classes, 10 orders, 13 families and 15 genera had a negative correlation with NDF digestibility, but only the relative abundance of *Pyramidobacter* had a positive correlation with NDF digestibility.

**Conclusion:**

There were substantial differences in NDF digestibility among the individual goats, and the NDF digestibility had significant correlation with the relative abundance of some ruminal microorganisms.

## INTRODUCTION

Fibre accounts for a high proportion of the diets of ruminants, and it is also an essential nutrient for ruminants. Therefore, the efficiency of feed utilization is significantly influenced by the dietary fibre digestibility in ruminants. It is well known that ruminants themselves can not secrete cellulolytic enzyme. Rather, it is ruminal microorganisms that play a crucial role in the degradation of fibre [1]. To enhance fibre digestibility, the vital step is to obtain a deep understanding of the microorganisms in the rumen.

In a previous report, *Fibrobacter succinogenes*, *Ruminococcus flavefacien*s, and *Ruminococcus albus* were considered to be the most important cellulolytic bacteria in the rumen [2]. However, many recent studies showed that *Ruminococcus* and *Fibrobacter succinogenes* had a very low relative abundance in the rumen [3,4], so we doubted that the role of these bacteria in the fibre degradation would be amplified. Further, many other important microorganisms associated with fibre digestion might have been ignored. Because previous studies of ruminal microorganisms were entirely dependent on culture techniques and small-scale sequencing, less than 10% of all microorganisms in the samples could be discovered.

Recently, next-generation sequencing technology had been extensively applied in the study of microbial ecology [4–6]. Compared to culture and fingerprint technologies, high-throughput sequencing can process millions of sequenced reads and obtain the biological information of many microorganisms simultaneously in samples. Inthis study, high-throughput sequencing was firstly applied to investigate the structure and composition of microorganisms of the rumen in goats that had the same genetics, age and management conditions but different neutral detergent fibre (NDF) digestibility.

## MATERIALS AND METHODS

### Animal care

The animal experimental procedures were approved by the Animal Policy and Welfare Committee of the Agricultural Research Organization of Sichuan Province, China, and were in accordance with the guidelines of the Animal Care and Ethical Committee of the Sichuan Agricultural University.

### Experimental animals and sampling

Nineteen castrated Boer crossbred goats (Jianchang black goat and Boer goat crossbreeds) with an average age of 1.5 years were used in this study. The average live body weight of the animals was 41.60±2.63 kg at the beginning of the study. All goats were fed the same total mixed rations containing 70% forage and 30% concentration (Table 1), and animals were fed at a restricted level of 3.5% body weight at 08:00, 13:00, and 18:00 hours. Each goat was housed in separate pens with free access to water. The trial lasted for 36 days, including 30 days of adaptation and 6 days of digestion period. Every day during the digestion period, all faeces were collected, and 10% of them were sampled randomly and then mixed with a 10% volume of 10% HCl for nitrogen fixation. At the same time, the daily feed intake and residual intake were recorded over six days for the subsequent calculation of nutritional composition and digestibility.

After digestion trials, the next morning before feeding, 50 mL of rumen contents from each goat were sampled using oral stomach tubes attached to an electric pump as previously described [7]. The rumen contents were repeatedly flapped on ice to ensure that the microbes associated with the feed particles were fully immersed in liquid. Then, the rumen contents were strained using four layers of gauze, and the rumen fluid was collected and aliquoted into 10-mL centrifuge tubes. Rumen liquid samples were sealed and stored at −80°C until DNA extraction.

### Analysis of samples and grouping

All samples of feed and faeces were dried in a forced-air oven at 65°C for 48 h to measure the dry matter (DM) and then ground to pass through a 40-mesh sieve. NDF and acid detergent fibre (ADF) in samples were determined using the filter bag technology without sodium sulphite, and expressed with residual ash. Ether extract (EE) was determined by Soxhlet extraction method, crude protein (CP) was determined by the Kjeldahl method, organic matter (OM) was measured in a muffle furnace at 550°C for 6 hours, and calcium and phosphorus were measured using an atomic absorption spectrophotometer [8].

The NDF digestibility was calculated as follows [5]:

NDF digestibility (%)=(A×B-C×D)/A×B×100

Where A was the feed intake computed as the amount of given feed minus the residual intake during a 6-day digestion period; B was the NDF concentration in the feed; C was the total amount of faeces based on DM in the 6-day digestion period; and D was the NDF concentration in faeces. The mean and standard deviation (SD) of NDF digestibility of all goats were calculated using Excel software. Then, standard deviations above and below the mean were used to group animals into high NDF digestibility (HFD, NDF digestibility>mean+ 0.5×SD) group and low NDF digestibility (LFD, NDF digestibility <mean−0.5×SD) group as the previous described method [9].

### DNA extraction

Total DNA was extracted from each rumen sample of the HFD and LFD groups using the TIANamp Bacteria DNA Kit (TIANGEN, Peking, China) according to the manufacturer’s guidelines as described before. Then, the quality and quantity of the DNA samples was determined through agarose electrophoresis and a Nanodrop 8000 Spectrophotometer (Thermo Fisher Scientific, Brisbane, Australia) respectively.

### Polymerase chain reaction amplification and sequencing

The universal primers of bacteria (341F:5′-CCTAYGGGRBGCA SCAG-3′ and A806R:5′--GGACTACHVGGGTWTCTAAT-3′) were used to amplify the V4 hyper variable regions of 16S rRNA [10]. All polymerase chain reaction (PCR) amplifications were performed in triplicate with 50 μL of reactions volume (PCR thermal cycler Model C1000, Bio-rad, Richmond, CA, USA) that consisted of 1.25 μL of each primer (10 μmol/L), 1 μL of 10 mmol/L dNTP Mixture, 5 μL of 10×ExTaq buffer (20 mmol/L Mg^2+^; TaKaRa Inc, Dalian, China), 1 μL of 50 ng/μL template DNA, 0.25 μL of 5 U/μL Taq DNA polymerase (Mg^2+^plus, Takara Inc., China) and distilled water to a final volume of 50 μL. The amplification was initiated with a denaturation at 94°C for 3 min, 30 cycles of denaturation at 94°C for 30 s, 58°C for 30 s, 72°C for 90 s, and a final extension at 72°C for 5 min. Finally, the three replicates of DNA extracted from each sample were mixed together.

The products were purified using a PCR Clean-Up system (Promega, Madison, WI, USA) with a purification kit (QIAGEN, Adelaide, Australia) and quantified using a QuantiFlour-ST fluorometer (Promega, USA) [8]. Subsequently, the amplicons of each reaction mixture were pooled into a single tube in equimolar ratios to generate the amplicon libraries. Before the samples were pooled with equal volumes, each amplicon library was firstly diluted to 1×10^9^ molecules/mL. Then the pooled amplicon libraries were diluted to 1×10^7^ molecules/mL. Finally, the samples were sent to Macrogen Inc. (Seoul, Korea) and sequenced on an Illumina MiSeq 300PE Sequencing Platform (Novogene, Beijing, China).

### Sequencing analysis

The QIIME pipeline software (version 1.8.0) was used to analyse the reads acquired from Macrogen Inc [11]. Low-quality sequences, such as sequences containing uncertain nucleotides, continuous three nucleotides with Q values less than 20 and unmatched barcode sequences, were removed. Chimeric sequences were removed using Usearch V7.0 based on the Uchime algorithm implemented in QIIME [12]. The clean and high-quality sequences were then clustered into operational taxonomic units (OTUs) based on 97% similarity. The most abundant sequence was selected as the representative for each OTU and then aligned against the Greengenes database (http://greengenes.lbl.gov) using PyNAST [13]. Taxonomic OTU assignments were performed using the RDP Classifier (http://rdp.cme.msu.edu) [4].

Alpha diversity indices (Shannon-Wiener, PD_whole_tree, Chao1 and The observed-species) were computed at the depth of 21,854 sequences. Beta diversity was visualized using principal co-ordinate analysis (PCoA), as measured using an unweighted UniFrac distance matrix. In addition, genera that were shared by all the samples were selected to create a heat map using the R version 3.0.2 software program [14]. All sequence data in the present study were deposited in the sequence read archive (SRA) of the NCBI database under the number PRJNA290544.

### Statistical analysis

An unpaired two-tailed t-test was performed usingSPSS Statistics software v. 19.0 (IBM, Armonk, NY, USA) to assess the differences of NDF digestibility and microbial relative abundance between the HFD and LFD groups. The results were shown as means±SD. The correlation analysis between the relative abundance of bacteria and NDF digestibility was performed. The significant and extremely significant levels were set at p<0.05 and p<0.01, respectively.

## RESULTS

### NDF digestibility

The average NDF digestibility of nineteen goats (Table 2) was 58.83%±5.36% (mean±SD). Then, HFD (NDF digestibility> mean+0.5×SD, n = 5) and LFD (NDF digestibility<mean−0.5× SD, n = 5) individuals were chosen. The difference in NDF digestibility between the groups was extremely significant (p< 0.001). There were no significant differences in the apparent digestibility of EE, CP, and ADF between the groups, but there were significant differences in the apparent digestibility of DM, OM between the groups. Comparisons of apparent digestibility of dietary nutrients of the two groups are shown in Supplementary Table S1.

### Analysis of the Illumina MiSeq sequencing data of the two groups

**Figure 1:**
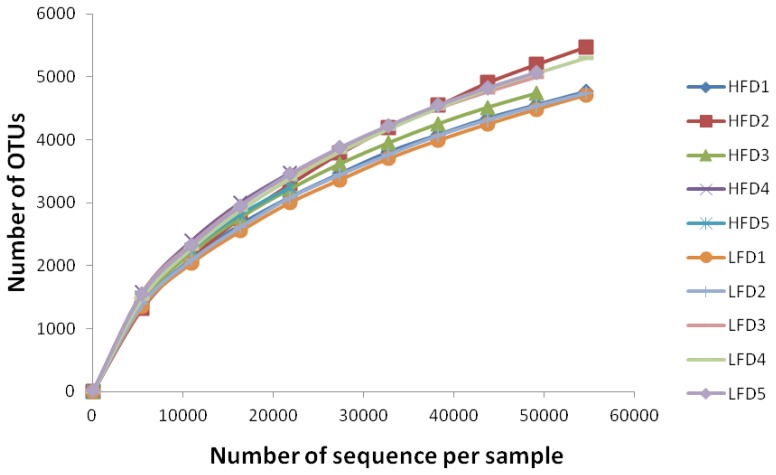
Rarefaction curves of each sample in the high neutral detergent fibre digestibility group and low neutral detergent fibre digestibility group.

*Sequencing depth and alpha diversity*: A total of 569,887 sequences were generated from the samples of all goats after quality control with a mean of 56,988±24,441 (mean±SD, n = 10). A total of 18,694 OTUs were identified at the 97% similarity level with a mean of 5,027±1,059 (mean±SD, n = 10). Distribution of valid sequences and OTUs were presented in Supplementary Figure S1 and S2. Alpha diversity was presented in Table 3. The Shannon-Wiener index, Chao I index, the Observed-species and PD_whole_tree had no significant difference between the LFD and HFD groups. To evaluate the depth of sampling, rarefaction curves produced from sequences and OTUs of bacteria sequencing were shown in Figure 1. The rarefaction curve showed that most samples were nearly asymptotic, which indicated that the depth of sequencing in the present study could cover most of the microorganisms in samples.

### Composition of the rumen bacteria

**Figure 2:**
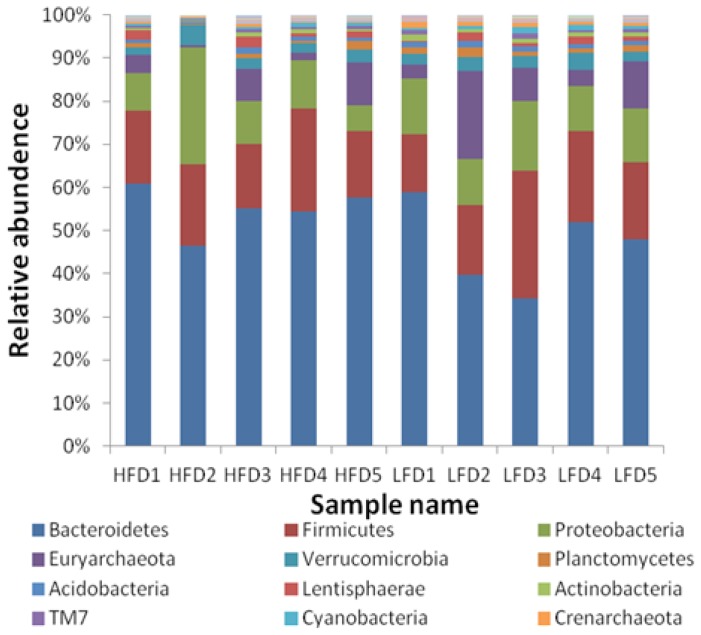
Taxonomic composition of the rumen bacterial communities on the phylum level.

After the taxonomic summary, all OTUs were classified as 43 phyla, with most of them (81.98%) belonging to bacteria and only 6.64% were archaea. The 17 phyla whose relative abundance was more than 0.1% in all samples were shown in Figure 2, and the other 26 phyla were not shown because of their low relative abundance. The relative abundance of Nitrospirae (p = 0.030) and Crenarchaeota (p = 0.011) in HFD group was significantly lower than that in LFD group. The three most predominant phyla were Bacteroidetes (48.57%±4.84% in HFD; 41.11%±10.07% in LFD), Firmicutes (15.93%±2.94% in HFD; 17.33%±5.85% in LFD) and Proteobacteria (11.37%± 8.21% in HFD; 11.08%±2.46%in LFD) in the two groups.

At the class level, Bacteroidia was the most predominant bacterium for all samples, and its average relative abundance reached 44.19%. The relative abundance of Gammaproteobacteria, Bacilli, Acidobacteria-6 and Nitrospira were significantly different, Thaumarchaeota and PBS-25 were extremely significantly different between the two groups. The most predominant order-level bacterium was Bacteroidales, which was one of the most important orders in phylum Bacteroidia. And the predominant bacterial family was Prevotellaceae (21.06%).

At the genus level, *Prevotella* was the most predominant taxa both in the two groups (20.98%). However, the relative abundance of *Prevotella* had no significant difference between the two groups. The comparisons of taxa relative abundance from the levels of phylum to genus were presented in Table 4.

### Shared genera

**Figure 3:**
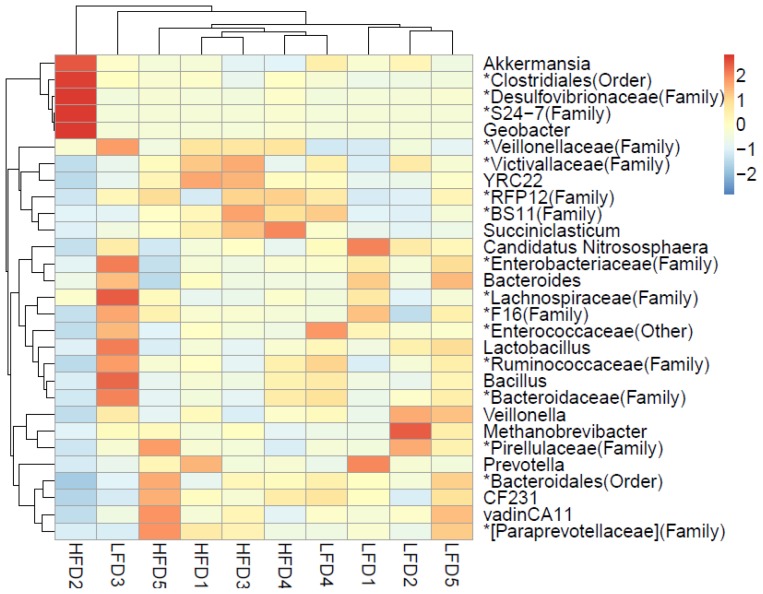
Heatmap of the shared genera between the high neutral detergent fibre digestibility group and low neutral detergent fibre digestibility group.

A total of 176 genera were shared by all goats, and these genera accounted for 85.20% of all taxa. There were 197 genera shared by the goats of HFD group and 227 genera shared by the goats of LFD group. These shared genera and phylogenetic placements among individuals were presented in a heatmap (Figure 3).

### Clustering dissimilarity analysis

**Figure 4:**
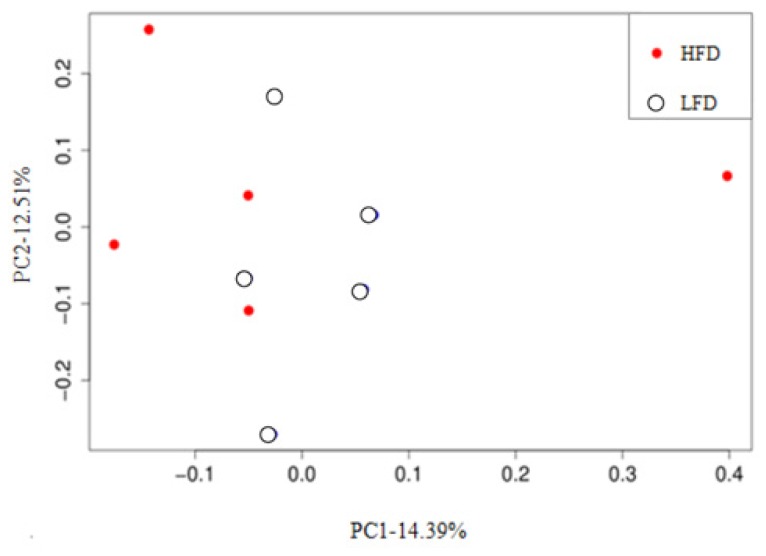
Principal coordinate analysis (PCoA) plots based on operational taxonomic units (OTUs, grouped at a 97% sequence similarity threshold) between high neutral detergent fibre digestibility group and low neutral detergent fibre digestibility group.

The unweighted UniFrac distance matrix among samples was measured, and the PCoA plot based on unweighted UniFrac distance metric was generated according to the OTU table (Figure 4). The closer the distance between points was, the higher similarity in the community structure was between two samples. The results showed that the bacterial community composition between groups and samples had no clear dissimilarity because the spots of the two groups were not clearly separated. Moreover, PC1 and PC2 components only contributed to 14.39% and 12.51% of the variability, respectively.

### Relationship between rumen microorganisms and NDF digestibility

The relative abundance of Crenarchaeota at the phylum level, Thaumarchaeota and PBS-25 at the class level, Nitrososphaerales, SBR1031 and PBS-25 (Class) at the order level, Nitrososphaeraceae, Sphingomonadaceae, *Lactobacillales (Other), Nitrospiraceae and SJA-101 at the family level, *Candidatus Nitrososphaera*, *Flavobacteriaceae (Family), *SJA-101 (Family), *Rhizobiaceae (Family), *Hyphomonadaceae (Family), and *Nitrosopumilus* at the genus level had extremely significantly negative relationship with NDF digestibility (p< 0.01). Furthermore, there were also one phylum-level, three class-level, seven order-level, eight family- level and nine genus-level bacteria had significant negative relationships with NDF digestibility (p<0.05). Only Pyramidobacter had positive correlation with NDF digestibility (p<0.05) (Table 5).

## DISCUSSION

The previous studies reported that the NDF digestibility in ruminants was mainly affected by physical and chemical pre-treatment methods of roughage, diet composition and additives [15–17]. It was generally believed that there was no significant difference in the digestibility of NDF in animals under the same conditions. However, in the present study, although experimental goats had the same genetic background, gender, and age were fed the same feed, obvious differences in NDF digestibility among individuals were found, with a wide range of 51.67% to 69.24%. To date, there have been few reports about the individual differences in NDF digestibility. Nevertheless, significant individual differences in residual feed intake (RFI) had been reported in a number of previous researches [18–20]. RFI was widely used to evaluate the feed utilization efficiency [21,22]. Feed utilization efficiency of ruminants depended jointly on the digestibility of various nutrients in feed, including NDF. Furthermore, the concentration of NDF in the diets of goats was usually more than 35%. Therefore, the individual differences in NDF digestibility in the present study were reasonable.

After the comparison analysis, it was found that difference in the NDF digestibility between the LFD (52.35±1.64) and HFD groups (65.85±2.56) (p<0.000) was significantly different in the present work. For ruminants, the rumen is the main site for the digestion of nutrients, especially for fibre. The digestion of fibre depends entirely on symbiotic ruminal microorganisms because the ruminants themselves cannot secrete cellulolytic enzymes [1,23]. Accordingly, the fundamental reason leading to the differences in NDF digestibility between the two groups in this study might due to the differences in the structure and composition of ruminal microorganisms. To prove this hypothesis, Illumina high-throughput sequencing was conducted to compare the microbiome communities between the two groups.

The high-throughput sequencing results indicated that the three predominant phyla in the rumens of all goats were Bacteroidetes, Firmicutes and Proteobacteria, which was consistent with many past studies in herbivores [6,14]. At the genus level, the relative abundance of *Prevotella* was the highest in all samples, and this result was similar to previous studies [24]. *Prevotella* was also shared by all of the samples and its relative abundance was not significantly difference between the two groups. This above results explained that the member of microbial flora between the two groups was not clearly different, which was also showed in the PCoA plot (Figure 4). It was probably due to the same genetic, age and diets of the experimental animals and the same rear conditions during the trials, which were regarded as the key factors that affected the member of microbial flora by the previous researchers [4, 6,14]. The difference of microbial flora was mainly presented in the relative abundance of bacteria.

Previous studies indicated that *Ruminococcus*, *Fibrobacter*, and *Clostridium* could produce a large amount of cellulase and hemicellulase *in vitro* [2]. Therefore, it was believed that these bacteria had an important role in fibre degradation and the higher abundance of them meant the higher fibre digestibility in the rumen. Thisopinion was partially consistent with the results of the present study, in which the relative abundance of *Ruminococcus* (0.60% in HFD, 0.37% in LFD), *Fibrobacter* (0.15% in HFD, 0.11% in LFD) and *Clostridium* (0.15% in HFD, 0.10% in LFD) were all higher in the HFD group than in the LFD group. However, the difference of their relative abundance between groups was not significant, and it was particularly worth noting that the relative abundance of these bacteria was very low. To improve the digestibility of fibre, previous studies had focused mainly on bacteria that could secrete cellulolytic enzymes [2,14]. However, in this study, the bacteria that had a negative relationship with NDF digestibility was flourished in the rumen. The degree of NDF digestion was likely determined by these “negative” microorganisms rather than cellulolytic bacteria because many of these “negative” microorganisms, such as Enterobacteriaceae, Lactobacillus, and Enterococcus had a much higher relative abundance than the cellulolytic bacteria mentioned above, and the differences of their relative abundance between groups were significant.

In the present study, Enterobacteriaceae was found to be the predominant family both in the two groups (Table 4), and its relative abundance in LFD (5.71%) was significantly higher than that in HFD (3.52%). The correlation between Enterobacteriaceae and NDF digestibility had not been reported. Some previous experiments showed that Enterobacteriaceae was a Gram-negative bacteria and contained opportunistic pathogens, such as *Escherichia coli* and Salmonella, which impaired gut health [25]. Additionally, the research from Sevcik showed that three species of Enterobacteriaceae, including *Enterobacter cloacae*, *E. asburiae*, and *Klebsiela pneumonia* were identified as the source of bovine saxitoxin, which is a paralytic toxin that can block sodium channels in nerves [26]. Up to now, most of studies on Enterobacteriaceae were pathological and pharmacological study and many bacteria in Enterobacteriaceae were considered unprofitable for the host. Accordingly, we inferred that Enterobacteriaceae would be disadvantage for NDF digestibility in ruminants. Nevertheless, this view had not been reported, and further study is needed to confirm it.

The previous study showed that *Lactobacillus* had a substantial role in lactate metabolism in rumen and there was a positive correlation between its relative abundance and the concentration of lactic acid [27]. In the current study, the relative abundance of *Lactobacillus* was higher in LFD than that in HFD, and the relative abundance of Lactobacillales increased with the decrease of NDF digestibility through the analysis of correlation. In the rumen, high concentrations of lactic acid led to an imbalance in rumen microorganism flora, and cellulolytic bacteria were more sensitive to the low pH conditions caused by lactic acid than other bacteria [28]. Furthermore, a research had indicated that subacute ruminal acidosis reduced the digestibility of NDF [29]. Therefore, it was probably due to a high concentration of lactate in the rumen of goats in the LFD group that suppressed the fibrolytic bacteria and further affected the NDF digestibility in this study.

In a previous study, a number of *Enterococcus* (*Enterococcus faecalis*, *Enterococcus durans*, *Enterococcus casseliflavus*) were identified as cellulase producers in the guts of insects and the colons of humans [30]. This study found the relative abundance of Enterococcaceae and the *Enterococcaceae (*Other*) (the bacteria that was not named in Enterococcaceae) in LFD was higher than that in HFD. Enterococcus is one of the mainly genus in Enterococcaceae, and there might some genus in Enterococcaceae have the negative relationship with NDF digestion. Therefore, whether the other bacteria in *Enterococcus* was related to the degradation of cellulose in the rumen needed further research.

There were also some bacteria whose relative abundance had a significant positive correlation with NDF digestibility, such as Veillonella and Bacteroides (Tables 4, 5). However, the relative abundance of these bacteria in rumen was very low. Therefore, their role in fibre digestion remains unknown and requires further research.

## CONCLUSION

The results obtained in this study indicated that the NDF digestibility was largely different among individual goats and there were significant differences in the structure of rumen microorganisms in goats with different NDF digestibility. Additionally, many bacteria had tight correlation with the digestibility of NDF. These results contribute further insights into the rumen bacterial structure under different NDF digestibility conditions and provide evidence for targeted improvement of dietary fibre digestibility in goats

## Supplementary Data



## Figures and Tables

**Table 1:** The composition and nutritional ingredients of the diet

Items	Content
Ingredients (%, DM basis)	
Alfalfa meal	35
Rice straw	35
Corn	2.67
Rice	8.33
Soybean meal	5.0
Wheat bran	13
Premix1)	0.5
NaCl	0.5
Total	100
Nutrition levels2)	
Metabolizable energy (MJ/kg)	7.7
Crude protein	10.21
Neutral detergent fibre	46.13
Calcium	0.92
Phosphorus	0.31

1) Premix provides the following nutrients per kg of the diet: Fe (as ferrous sulfate) 30 mg; Cu (as copper sulfate) 10 mg; Zn (as zinc sulfate) 50 mg; Mn (as manganese sulfate) 60 mg; Vit A 2,937 IU; Vit D 343 IU dry matter; Vit E 30 IU.

2) Nutrition levels are values of measurement except that ME is a value from a calculation.

**Table 2:** The NDF digestibility of the diets of 19 Boer goats

Goats number	NDF digestibility (%)
1	51.24
2	51.48
3	51.67
4	52.11
5	55.23
6	56.96
7	57.03
8	57.43
9	57.69
10	57.72
11	58.14
12	59.35
13	61.52
14	61.03
15	62.55
16	64.60
17	65.52
18	67.33
19	69.24
Mean	58.83
SD	5.36

NDF, neutral detergent fibre; SD, standard deviation.

**Table 3:** The alpha diversity index (calculated at a depth of 21,854 sequences) of microbial communities from LFD and HFD groups (mean±SD, n = 5)

Items	HFD	LFD	p value
PD_whole_tree	223.5±5.9	223.1±15.5	0.966
Chao 1	5,761.2±692.3	6,008.7±297.5	0.484
Shannon	8.7±0.4	8.7±0.4	0.790
The observed-species	3,267±692	6,009±297	0.484

LFD, low neutral detergent fibre digestibility group; HFD, high neutral detergent fibre digestibility group; SD, standard deviation.

**Table 4:** The microorganisms with significant differences between HFD and LFD groups from phylum to genus

Taxa		Relative abundance (%)		

		HFD (n = 5)	LFD (n = 5)	p value
Phylum	Crenarchaeota	0.335±0.178	0.850±0.299	0.011
	Nitrospirae	0.047±0.013	0.089±0.033	0.030
Class	Gammaproteobacteria	4.934±1.022	7.693±2.196	0.034
	Bacilli	3.542±1.080	5.945±1.767	0.032
	Thaumarchaeota	0.323±0.179	0.836±0.274	0.008
	Acidobacteria-6	0.349±0.157	0.562±0.063	0.023
	Nitrospira	0.047±0.013	0.089±0.033	0.030
	PBS-25	0.000	0.002±0.001	0.000
Order	Enterobacteriales	3.559±0.780	5.756±1.626	0.026
	Lactobacillales	2.514±0.681	4.341±1.038	0.011
	Nitrososphaerales	0.311±0.172	0.785±0.238	0.007
	iii1–15	0.331±0.145	0.538±0.080	0.023
	Pseudomonadales	0.332±0.082	0.459±0.092	0.050
	Thiotrichales	0.134±0.030	0.218±0.032	0.003
	SBR1031	0.052±0.032	0.161±0.084	0.027
	[Marinicellales]	0.043±0.021	0.096±0.042	0.035
	MND1	0.032±0.024	0.105±0.028	0.002
	Nitrospirales	0.047±0.013	0.089±0.033	0.030
	[Entotheonellales]	0.004±0.006	0.026±0.018	0.034
	*PBS-25 (Class)	0.000	0.002±0.001	0.001
Family	Enterobacteriaceae	3.559±0.780	5.756±1.626	0.026
	Bacteroidaceae	2.081±0.344	3.058±0.501	0.007
	Enterococcaceae	1.477±0.303	2.129±0.521	0.042
	Lactobacillaceae	0.800±0.311	1.869±0.772	0.021
	Nitrososphaeraceae	0.311±0.172	0.785±0.238	0.007
	*iii1–15 (Order)	0.270±0.118	0.419±0.046	0.029
	Sphingomonadaceae	0.225±0.091	0.354±0.013	0.014
	Piscirickettsiaceae	0.134±0.030	0.218±0.032	0.003
	Pseudomonadaceae	0.063±0.025	0.117±0.032	0.019
	[Marinicellaceae]	0.043±0.021	0.096±0.042	0.035
	*MND1 (Order)	0.032±0.024	0.105±0.028	0.002
	*Rhizobiales (Order)	0.032±0.017	0.098±0.054	0.031
	Ellin517	0.034±0.027	0.071±0.011	0.023
	*Lactobacillales (Other)	0.029±0.014	0.059±0.014	0.010
	Nitrospiraceae	0.016±0.009	0.051±0.026	0.022
	*Lactobacillales (Order)	0.014±0.007	0.032±0.016	0.050
	SJA-101	0.008±0.009	0.037±0.016	0.008
	Hyphomonadaceae	0.009±0.008	0.022±0.006	0.022
	Saccharospirillaceae	0.000	0.001±0.001	0.044
Genus	*Enterobacteriaceae (Family)	3.524±0.770	5.710±1.623	0.026
	*Enterococcaceae (Other)	1.182±0.221	1.750±0.408	0.026
	*Lactobacillus*	0.800±0.311	1.869±0.772	0.021
	*Bacteroides*	0.915±0.168	1.299±0.315	0.040
	*Veillonella*	0.501±0.159	0.804±0.219	0.037
	*CandidatusNitrososphaera*	0.308±0.171	0.776±0.244	0.008
	5–7N15	0.334±0.095	0.561±0.169	0.031
	*Kaistobacter*	0.172±0.070	0.269±0.042	0.028
	*Piscirickettsiaceae (Family)	0.133±0.030	0.217±0.032	0.003
	*Pseudomonas*	0.054±0.013	0.098±0.035	0.032
	* [Marinicellaceae] (Family)	0.043±0.021	0.096±0.042	0.035
	*Flavobacteriaceae (Family)	0.030±0.015	0.078±0.037	0.029
	*Ellin517 (Family)	0.034±0.027	0.071±0.011	0.023
	*SJA-101 (Family)	0.008±0.009	0.037±0.016	0.008
	*Pyramidobacter*	0.028±0.016	0.008±0.006	0.034
	*Rhizobiaceae (Family)	0.004±0.005	0.028±0.007	0.000
	*Hyphomonadaceae (Family)	0.007±0.007	0.022±0.006	0.007
	*Nitrosopumilus*	0.002±0.004	0.025±0.012	0.004
	*Paraprevotella*	0.002±0.002	0.007±0.004	0.046
	*Cenarchaeaceae (Family)	0.001±0.002	0.006±0.004	0.049
	*Dyadobacter*	0.000	0.001±0.001	0.048
	*Saccharospirillum*	0.000	0.001±0.001	0.044

HFD, high neutral detergent fibre digestibility group; LFD, low neutral detergent fibre digestibility group.

Taxa that could not be assigned to a certain classification but still had significant differences between groups were displayed using the highest taxonomic level at which they could be assigned. In addition, the levels are shown in parentheses and with the superscript “*”. The same below.

**Table 5:** The correlation between the relative abundance of microorganisms and NDF digestibility

	Taxa	R	pvalue
Phylum	Crenarchaeota	−0.891	0.001
	Nitrospirae	−0.733	0.016
Class	Bacilli	−0.491	0.15
	Thaumarchaeota	−0.891	0.001
	Acidobacteria-6	−0.745	0.013
	Nitrospira	−0.733	0.016
	PBS-25	−0.847	0.002
Order	Enterobacteriales	−0.673	0.033
	Lactobacillales	−0.685	0.029
	Nitrososphaerales	−0.891	0.001
	iii1–15	−0.661	0.038
	Thiotrichales	−0.745	0.013
	SBR1031	−0.879	0.001
	MND1	−0.721	0.019
	Nitrospirales	−0.721	0.016
	[Entotheonellales]	−0.693	0.026
	*PBS-25 (Class)	−0.847	0.002
Family	Enterobacteriaceae	−0.673	0.033
	Bacteroidaceae	−0.685	0.029
	Nitrososphaeraceae	−0.891	0.001
	*iii1–15 (Order)	−0.624	0.054
	Sphingomonadaceae	−0.806	0.005
	Piscirickettsiaceae	−0.745	0.013
	*MND1 (Order)	−0.721	0.019
	Ellin517	−0.661	0.038
	*Lactobacillales (Other)	−0.855	0.002
	Nitrospiraceae	−0.806	0.005
	*Lactobacillales (Order)	−0.709	0.022
	SJA-101	−0.818	0.004
	Hyphomonadaceae	−0.685	0.029
Genus	*Enterobacteriaceae (Family)	−0.673	0.033
	Enterococcaceae (Other)	−0.721	0.019
	*Candidatus Nitrososphaera*	−0.891	0.001
	*Piscirickettsiaceae (Family)	−0.733	0.016
	*Pseudomonas*	−0.709	0.022
	*Flavobacteriaceae (Family)	−0.782	0.008
	*Ellin517 (Family)	−0.661	0.038
	SJA-101 (Family)	−0.818	0.004
	*Pyramidobacter*	0.661	0.038
	*Rhizobiaceae (Family)	−0.818	0.004
	*Hyphomonadaceae (Family)	−0.806	0.005
	*Nitrosopumilus*	−0.804	0.005
	*Paraprevotella*	−0.657	0.039
	*Cenarchaeaceae (Family)	−0.632	0.05
	*Dyadobacter*	−0.649	0.042

NDF, neutral detergent fibre.
