# Supplementary material for: Relationship between the structure and composition of rumen microorganisms and the digestibility of neutral detergent fibre in goats
Source: Asian-Australas J Anim Sci. 2018 Jul 26;32(1):82–91. doi: 10.5713/ajas.18.0043 (PMC6325412; doi:10.5713/ajas.18.0043)

Supplementary information for:

Study on the relationship between the structure and composition of rumen microorganisms and the digestibility of neutral detergent fibre in goats

Kaizhen Liu[a], Lizhi Wang[a,*], Tianhai Yan[b], Zhisheng Wang[a], Bai Xue[a], Quanhui Peng[a]

[a]Institute of Animal Nutrition, Key Laboratory of Bovine Low-Carbon Farming and Safe Production, Sichuan Agricultural University, Ya'an, Sichuan 625014, China

[b]Agri-Food and Biosciences Institute, Hillsborough, Co. Down BT26 6DR, UK

*__Corresponding Author:__ Lizhi Wang, Institute of Animal Nutrition, Sichuan Agricultural University, Ya'an, Sichuan, 625014, China. Tel: 0086-835-2885165; Fax: 0086-835-2885065; E-mail: wanglizhi08@aliyun.com.

Table S1 Apparent digestibility of dietary nutrients in two groups

Items	Apparent digestibility（%）	
	HFD（n=5）	LFD（n=5）
DM	$59.80±1.02^{a}$	$55.88±1.38^{c}$
OM	$62.74±1.57^{a}$	$58.60±1.68^{c}$
EE	$73.95±9.38^{a}$	$73.72±5.21^{a}$
CP	$66.17±1.82^{a}$	$62.25±3.44^{a}$
NDF	$52.35±1.64^{a}$	$65.85±2.56^{c}$
ADF	46.16＋14.55 ^{a}	$52.06±11.74^{a}$

Note: In the same row, values with adjacent letter superscripts indicated significant difference (P<0.05), and with no adjacent superscript letters indicated dramatically significant difference (P<0.01), while same small letter superscripts indicated no significant difference (P>0.05),

Fig. S1. Distribution of valid sequences per sample

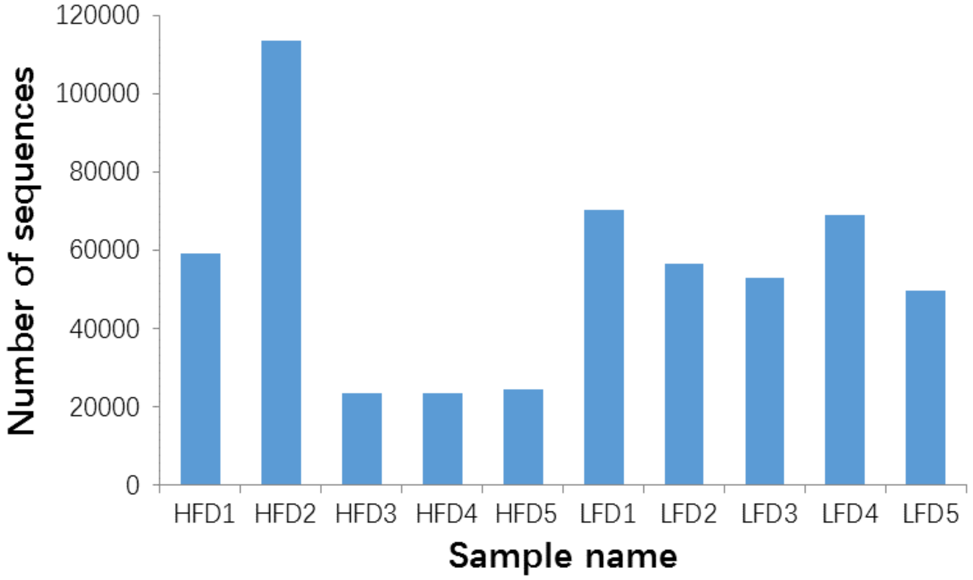

Fig. S2. Distribution of OTU per sample

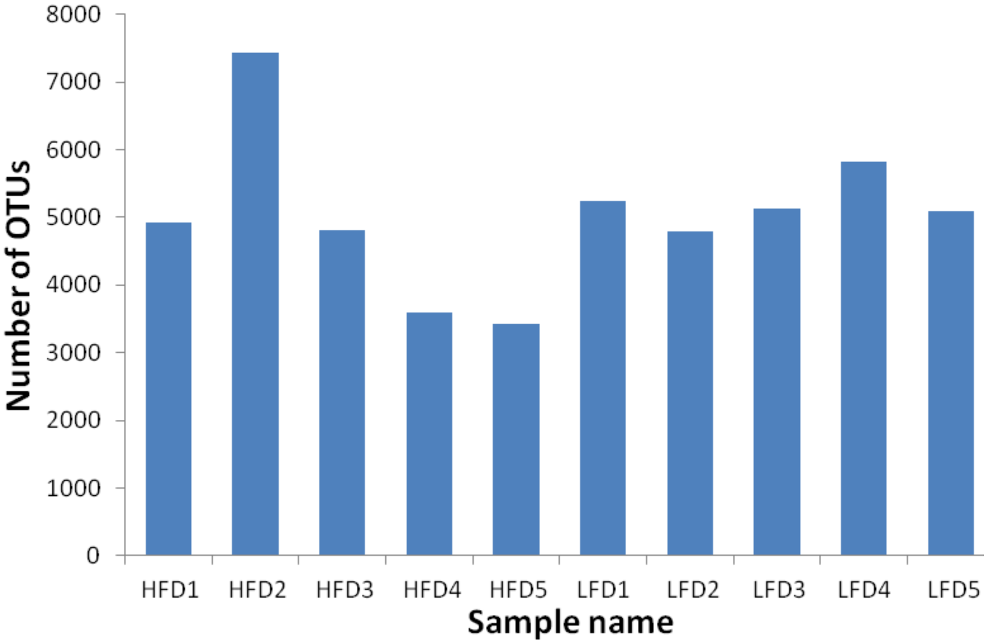

Supplement: Supplementary file 1 [file ajas-18-0043-supplmentary.pdf]
